# Large‐scale Whole‐Exome Sequencing Defines the Protein‐Coding Architecture of Retinal Structure, Visual Function, and Major Blinding Diseases

**DOI:** 10.1002/advs.76582

**Published:** 2026-07-24

**Authors:** Jianqing Li, Yijun Ge, Jingxiao Du, Liu Yang, Bangsheng Wu, Chenyue Hang, Mengxi Shen, Yimin Mao, Ting Zhang, Qiyu Bo, Tianyuan Zhao, Yutian Jiao, Yazhi Wang, Shunxiang Gao, Jieqiong Chen, Junran Sun, Tong Li, Huixun Jia, Yang Dou, Wei Cheng, Xiaoling Wan, Jintai Yu, Xiaodong Sun, Bai Lu

**Affiliations:** ^1^ Department of Ophthalmology Shanghai General Hospital Shanghai Jiao Tong University School of Medicine Shanghai China; ^2^ National Clinical Research Center for Eye Diseases Shanghai China; ^3^ Department of Ophthalmology The First Affiliated Hospital of Soochow University Suzhou China; ^4^ Department of Neurology and National Center For Neurological Disorders Huashan Hospital State Key Laboratory of Brain Function and Disorders MOE Frontiers Center For Brain Science Shanghai Academy of Natural Sciences New Cornerstone Science Laboratory Shanghai Medical College Fudan University Shanghai China; ^5^ Department of Ophthalmology Bascom Palmer Eye Institute University of Miami Miller School of Medicine Miami Florida USA; ^6^ Institutes of Biomedical Sciences Fudan University Shanghai China; ^7^ Shanghai Engineering Center For Visual Science and Photomedicine Shanghai China; ^8^ Shanghai Key Laboratory of Fundus Diseases Shanghai China; ^9^ School of Pharmaceutical Sciences IDG/McGovern Institute for Brain Research Tsinghua University Beijing China; ^10^ Institute of Science and Technology for Brain‐Inspired Intelligence Fudan University Shanghai China; ^11^ Shanghai Gene Therapy Center Shanghai China; ^12^ Shanghai Academy of Natural Sciences Shanghai China

**Keywords:** genetic pleiotropy, protein‐coding variation, rare coding variants, retinal phenotypes, whole‐exome sequencing

## Abstract

The retina is an accessible extension of the central nervous system, yet the protein‐coding architecture linking retinal structure, visual function, and major blinding diseases remains poorly defined. Here, using large‐scale whole‐exome sequencing data from 356,982 UK Biobank participants, exome‐wide gene‐based tests of rare coding variants and single‐variant analyses of common coding variants are performed. A total of 22 significant rare‐variant gene‐based associations involving 16 genes are identified, including 12 novel genes, 10 of which are independently supported in the All of Us cohort (N = 245,388). Single‐variant analyses identify 243 independent common coding variants in 126 genes, including 24 novel genes. *CFI*, *C3*, and *RIOX1* show associations across retinal structure, visual function, and disease phenotypes, supporting cross‐domain pleiotropy. Among the disease‐associated genes, the novel gene *FYB2* is prioritized for experimental validation, supported by retinal pigment epithelium (RPE)‐related expression evidence and clinical relevance in Cox analyses. *FYB2* knockdown aggravates barrier dysfunction in human induced RPE (iRPE) cells, supporting a potential role in diabetic retinopathy. These findings define the protein‐coding architecture of retinal phenotypes, and support shared genetic links across retinal structure, visual function, and disease. The identified genes provide candidate targets for mechanistic investigation in blinding retinal disorders.

## Graphical abstract

Large‐scale whole‐exome sequencing in 356,982 UK Biobank participants defines the protein‐coding architecture of retinal structure, visual function, and major blinding diseases. Pleiotropic genes, including *CFI*, *C3*, and *RIOX1*, bridge multiple retinal phenotypes, while experimental validation of *FYB2* implicates RPE barrier dysfunction, providing candidate targets for mechanistic investigation and future therapeutic exploration in blinding retinal diseases.

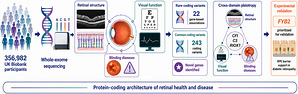

## 1 Introduction

The retina is a highly accessible extension of the central nervous system (CNS) and provides a window into systemic health [1, 2]. Retinal structure is tightly linked to visual function, and disruption of this structure‐function architecture contributes to major blinding diseases. Vision impairment is a major global health concern, affecting over 2.2 billion people worldwide according to the World Health Organization in 2023 [3]. Among the leading causes of blindness, retinal diseases such as age‐related macular degeneration (AMD), glaucoma, and diabetic retinopathy (DR) are major contributors to irreversible vision loss [4]. Although recent studies have identified genetic variants associated with retinal traits and diseases [5, 6, 7, 8, 9, 10], the shared genetic architecture linking retinal structure, visual function, and major blinding diseases remains poorly defined.

Genome‐wide association studies (GWAS) have substantially advanced our understanding of retinal genetics, identifying hundreds of loci associated with retinal structure [11], visual function [12], and major blinding diseases such as AMD [5, 6], glaucoma [7, 8], and DR [9, 10]. However, most GWAS signals fall within non‐coding regions and explain only a small proportion of heritability [13]. Whole‐exome sequencing (WES) complements GWAS by directly interrogating protein‐coding regions. Recent large‐scale WES studies have demonstrated the value of coding variation for clarifying disease mechanisms and therapeutic targets [14, 15], and have begun to clarify the coding architecture of retinal diseases [16, 17, 18]. However, no large‐scale exome‐wide study has systematically interrogated shared coding variation across retinal structure, visual function, and major blinding diseases. With exome data from over 500,000 participants and extensive ocular phenotyping spanning OCT imaging, visual function, and disease endpoints, the UK Biobank (UKB) now provides a unique opportunity to address this gap within a unified coding‐variant framework.

**FIGURE 1:**
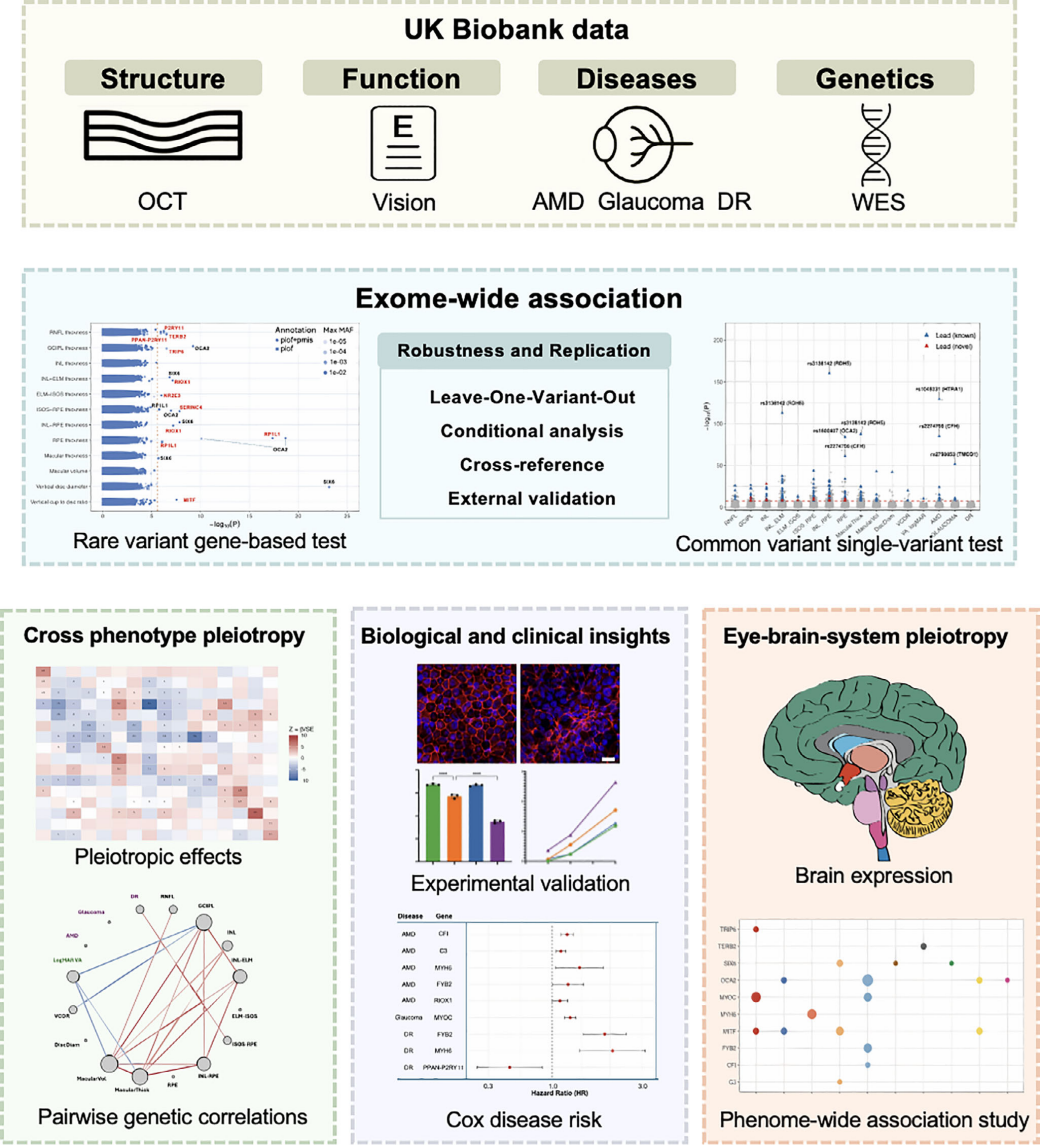
Overview of the study design and analytical framework. The study leveraged UK Biobank resources spanning retinal structure (optical coherence tomography, OCT), visual function, major vision‐threatening diseases (age‐related macular degeneration, glaucoma, and diabetic retinopathy), and whole‐exome sequencing (WES). Exome‐wide association analyses incorporated rare‐variant gene‐based tests, common‐variant single‐variant tests, and a series of robustness evaluations including leave‐one‐variant‐out analysis, conditional analysis, cross‐referencing, and external validation. Cross‐phenotype pleiotropy was assessed through pleiotropic effect mapping and pairwise rare‐variant genetic correlations across retinal structure, function, and disease. Biological and clinical interpretation was further supported by experimental validation and Cox regression analysis of disease risk. Eye‐brain‐system pleiotropy was further evaluated using cross‐tissue brain expression profiling and phenome‐wide association analyses, extending retinal genetic insights into broader neurobiological and systemic contexts.

Here, we leveraged the UKB WES data to perform exome‐wide analyses across multidimensional retinal phenotypes, including OCT‐derived structural traits, visual function measures, and three major vision‐threatening diseases: AMD, glaucoma, and DR. By integrating rare‐variant gene‐based analyses and common coding single‐variant analyses, we aimed to define the protein‐coding architecture linking retinal structure, visual function, and disease within a unified framework. We further performed integrative analyses and functional validation to characterize biologically and clinically relevant coding‐variant signals in the retina and explore their broader relevance. An overview of the study design is shown in Figure 1.

## 2 Results

### 2.1 Population Characteristics and Phenotype Overview

A total of 356,982 unrelated Caucasian individuals from the UKB were included after quality control (Methods). The mean age at enrollment was 57 ± 8.0 years, and 54% of participants were female (Table S1).

We analyzed three categories of retinal phenotypes: (1) structural traits, comprising 12 OCT‐derived phenotypes of retinal layer thickness, macular morphology traits, and optic nerve head parameters; (2) a functional trait, logarithm of the minimum angle of resolution of visual acuity (logMAR VA); and (3) three vision‐threatening retinal diseases, including AMD, glaucoma, and DR. Detailed sample sizes and corresponding UKB data fields for each phenotype are summarized in Table S2.

### 2.2 Exome‐Wide Rare‐Variant Analysis Identifies Genes for Retinal Structure, Function, and Disease

**FIGURE 2:**
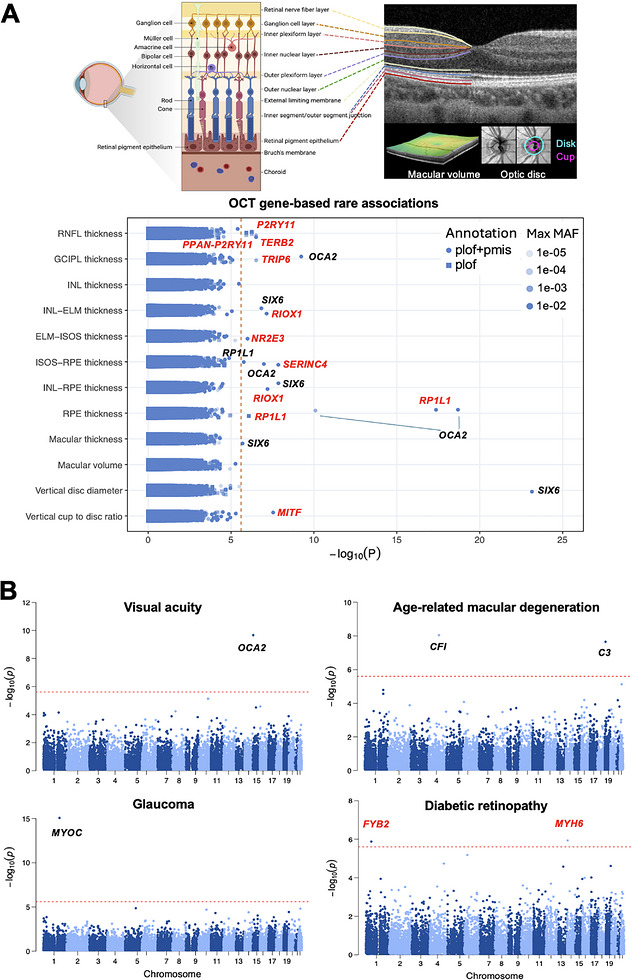
Exome‐wide rare‐variant association results for retinal phenotypes. (A) Top panel: Schematic illustrations of the retinal laminar architecture and representative optical coherence tomography (OCT) B‐scans, highlighting key structural layers and derived quantitative traits for macular volume and optic disc parameters. Bottom panel: Gene‐based associations between rare variants and retinal structural traits. The x‐axis denotes ‐log_10_(P) values from SAIGE‐GENE+ tests, and the y‐axis lists 12 OCT traits. Point shape indicates the functional annotation category (predicted loss‐of‐function [plof] + predicted missense [pmis] or plof), and color intensity encodes the maximum minor allele frequency (MAF) threshold. The vertical dashed line marks the exome‐wide significance threshold (*p* < 2.5 × 10^−6^). Genes surpassing this threshold are labelled, with novel associations highlighted in red. (B) Manhattan plots of gene‐based rare‐variant associations for visual acuity (logarithm of the minimum angle of resolution, logMAR VA), age‐related macular degeneration, glaucoma, and diabetic retinopathy. The x‐axis denotes genomic position, and the y‐axis denotes ‐log_10_(P) values. The red dashed line marks the exome‐wide significance threshold (*p* < 2.5 × 10^−6^). Genes exceeding this threshold are labelled, with novel associations highlighted in red.

To delineate the contribution of rare coding variation (minor allele frequency [MAF] < 1%) to retinal structure, visual function, and blinding diseases, we conducted exome‐wide gene‐based association analyses. In total, we identified 24 significant associations at the exome‐wide threshold (*p* < 2.5 × 10^−6^), of which 22 remained significant after global false discovery rate (FDR) correction, involving 16 genes across 16 retinal phenotypes (Figure 2A and Table S3). OCT structural traits accounted for 18 associations, including 11 novel gene‐trait signals. Several genes showed multilayer effects: *SIX6* across four layers, *OCA2* across three layers, and *RIOX1* and *RP1L1* across two layers. For visual acuity, *OCA2* showed a significant gene‐level association (*p* = 2.16 × 10^−10^). Notably, *OCA2* also influences three retinal layers (GCIPL, ISOS‐RPE, RPE), highlighting a structure‐function link. Among disease phenotypes, we identified five significant rare‐variant associations. For AMD, both *CFI* (*p* = 8.96 × 10^−9^) and *C3* (*p* = 2.20 × 10^−8^) reached exome‐wide significance under the missense+LoF mask, consistent with their established roles in complement dysregulation [19, 20]. For glaucoma, *MYOC* showed strong associations (*p* = 8.66 × 10^−16^ under missense+LoF; *p* = 6.79 × 10^−15^ under LoF only). For DR, *MYH6* (*p* = 1.16 × 10^−6^) and *FYB2* (*p* = 1.32 × 10^−6^) also reached significance (Figure 2B).

The overall distribution of test statistics showed no systematic inflation, as illustrated by the Q‐Q plots (Figure S1). Leave‐one‐variant‐out (LOVO) analyses clarified the variant architecture underlying each gene‐level association (Methods and Table S4, Figure S2). LOVO pinpointed the key rare variants driving most associations, such as *CFI* and *C3* in AMD and *MYOC* in glaucoma. At the same time, LOVO also highlighted genes whose signals were supported by multiple contributing variants, including *TERB2* for RNFL thickness and *OCA2* and *RP1L1* for RPE thickness.

Conditional analyses confirmed that most signals were independent of common variants (Table S5), although attenuation of signals at *OCA2*, *SIX6*, *C3*, and *RP1L1* suggested partial overlap with neighboring GWAS loci, indicating that both rare and common variants may contribute to the genetic architecture at these regions. Notably, conditioning on the *RP1L1* signal in the RPE revealed an additional independent rare‐variant association in *MCMDC2*, indicating a secondary signal within the *RP1L1*‐*MCMDC2* locus.

Cross‐referencing with Genebass, the GWAS Catalog, and PubMed classified 12 associations as novel, 3 as previously reported, and the remainder as extended associations (Table S3), indicating the robustness of our analysis and the reliability of the detected signals. Previously reported associations included *C3*‐AMD, *CFI*‐AMD, and *MYOC*‐glaucoma in prior exome sequencing studies; notably, *MYOC* also reached significance in Genebass under the missense mask. Among extended associations, *SIX6*, a well‐established glaucoma gene, demonstrated association with DiscDiam, a parameter strongly correlated with glaucoma risk. Both *SIX6* and *OCA2* also showed broader multilayer involvement than documented in the GWAS Catalog. Moreover, although the albinism‐related variant *OCA2*: c.1327G>A has been linked to reduced visual acuity [21], our result reflects a gene‐level association between *OCA2* and visual acuity.

For external replication, we utilized the All by All resource of the All of Us Research Program, which provides gene‐based rare‐variant associations across multiple ancestries. Consistent with our primary findings, *CFI* showed a significant association with AMD (*p* = 7.23 × 10^−8^) (Table S3). The *MYOC*‐glaucoma association was also externally replicated. Beyond these known genes, 10 of the 12 novel genes showed significant associations with clinically relevant blindness‐ and vision‐related phenotypes in All of Us, including “blindness and low vision” and “disorders of the optic nerve and visual pathways,” with only *P2RY11* and *MITF* lacking external annotation. Several additional genes also exhibited cross‐phenotype support, such as *C3* with blindness and low vision. Taken together, these analyses provide independent external support for the rare‐variant associations identified in our study.

We further leveraged newly released ocular phenotypes from the UKB, derived from OCT and color fundus imaging (released November 2025), as an internal phenotypic validation set. Specifically, we tested whether rare‐variant carriers of *SIX6* and *MITF*, the two genes associated with optic disc morphology in the discovery analysis, exhibited consistent effects on newly available optic disc traits, including optic disc major and minor axis length and cup‐disc ratio. In parallel, we assessed whether rare‐variant carriers of the ten genes associated with OCT structural traits showed concordant associations with the newly released central subfield thickness measure (Table S6). Robust and directionally consistent associations were observed for *SIX6* and *MITF* across optic disc‐related traits, while *OCA2* showed the strongest carrier‐based association with central subfield thickness (FDR‐adjusted *p* = 0.0028).

### 2.3 Exome‐Wide Common‐Variant Analysis Reveals Additional Loci for Retinal Traits

**FIGURE 3:**
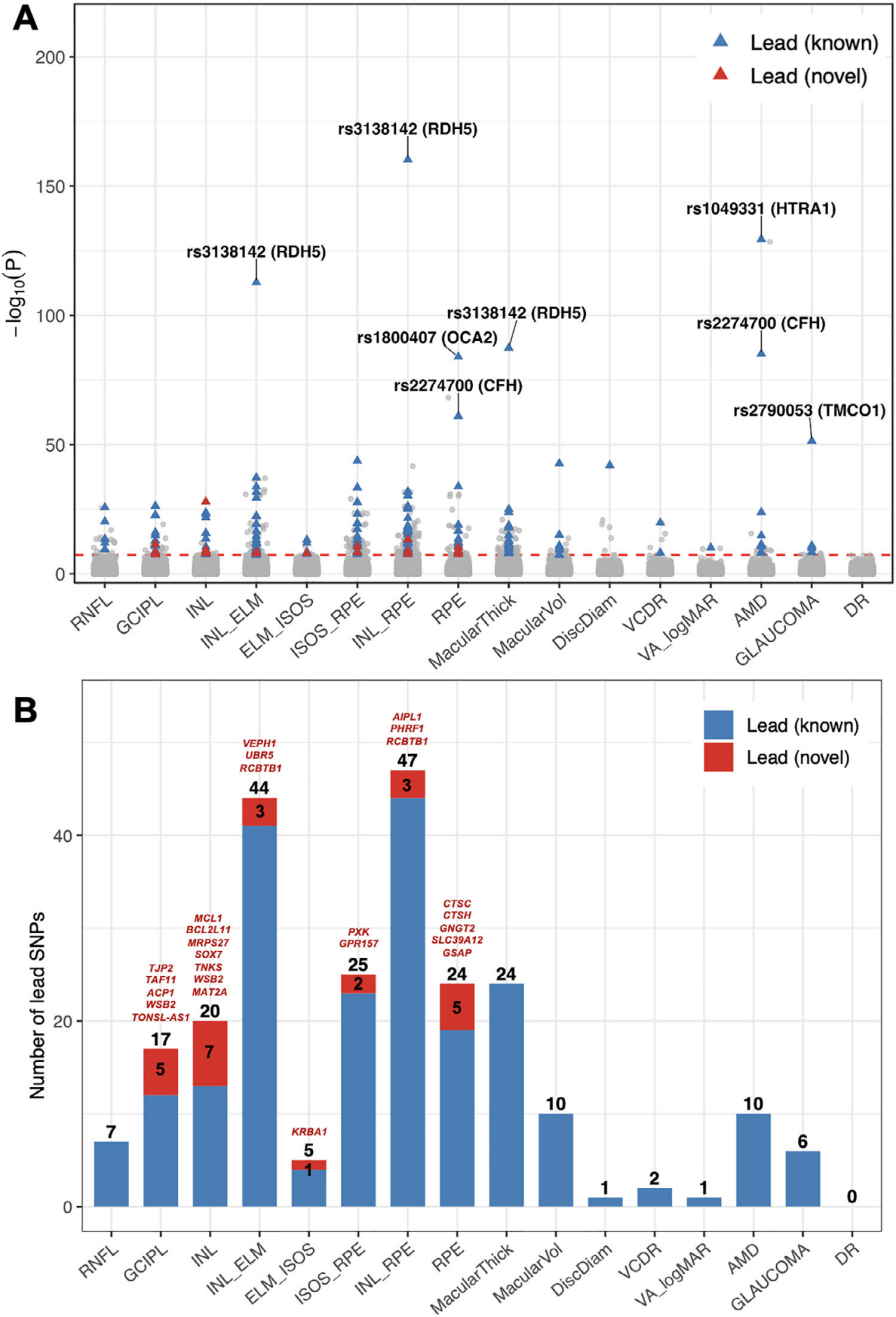
Exome‐wide common‐variant association results for retinal phenotypes. (A) Manhattan plot of single‐variant associations between common variants and 16 retinal phenotypes, including 12 optical coherence tomography (OCT) structural traits, visual acuity (logarithm of the minimum angle of resolution, logMAR VA), age‐related macular degeneration (AMD), glaucoma, and diabetic retinopathy (DR). The *x*‐axis denotes phenotypes, and the y‐axis denotes ‐log_10_(P) values. The red dashed line marks the genome‐wide significance threshold (*p* < 5 × 10^−8^). Each point represents a variant, with lead single‐nucleotide polymorphisms (SNPs) indicated by triangles. Novel lead SNPs are highlighted in red. For clarity, only loci with ‐log_10_(*P*) > 50 are labelled. (B) Stacked bar chart showing the number of lead coding SNPs reaching genome‐wide significance after linkage‐disequilibrium (LD) clumping for each phenotype. Bars are divided into novel (red) and known (blue) associations. Numbers above the bars indicate the total number of lead SNPs per phenotype. The gene symbols corresponding to novel lead SNPs are annotated directly above the bars. No lead SNP was identified for DR.

To complement the rare‐variant analyses, we performed WES‐based single‐variant association tests for common coding variants. Whereas rare variants implicated a small number of genes, common‐variant analyses identified a substantially larger number of association signals. Single‐variant association analyses for common variants (MAF ≥ 1%) were performed for the same phenotypes. In total, 584 genome‐wide significant associations (*p* < 5 × 10^−8^) were identified across all phenotypes except DR, and all remained significant after global multi‐phenotype correction (Methods and Table S7). After LD clumping, 243 independent coding variants mapped to 126 genes (Figure 3A).

Cross‐referencing with the GWAS Catalog identified 24 novel genes across 26 retinal‐trait associations, all involving OCT‐derived structural phenotypes (Figure 3B and Table S8). Among these, *MRPS27*, *SOX7*, and *GNGT2* showed multi‐layer associations. Although previously linked to photoreceptor‐layer thickness, each gene exhibited additional layer‐specific effects in our analysis: *MRPS27* and *SOX7* were associated with inner nuclear layer (INL) thickness, and *GNGT2* with retinal pigment epithelium (RPE) thickness.

### 2.4 Cross‐Phenotype Pleiotropy of Rare‐Variant Genes

**FIGURE 4:**
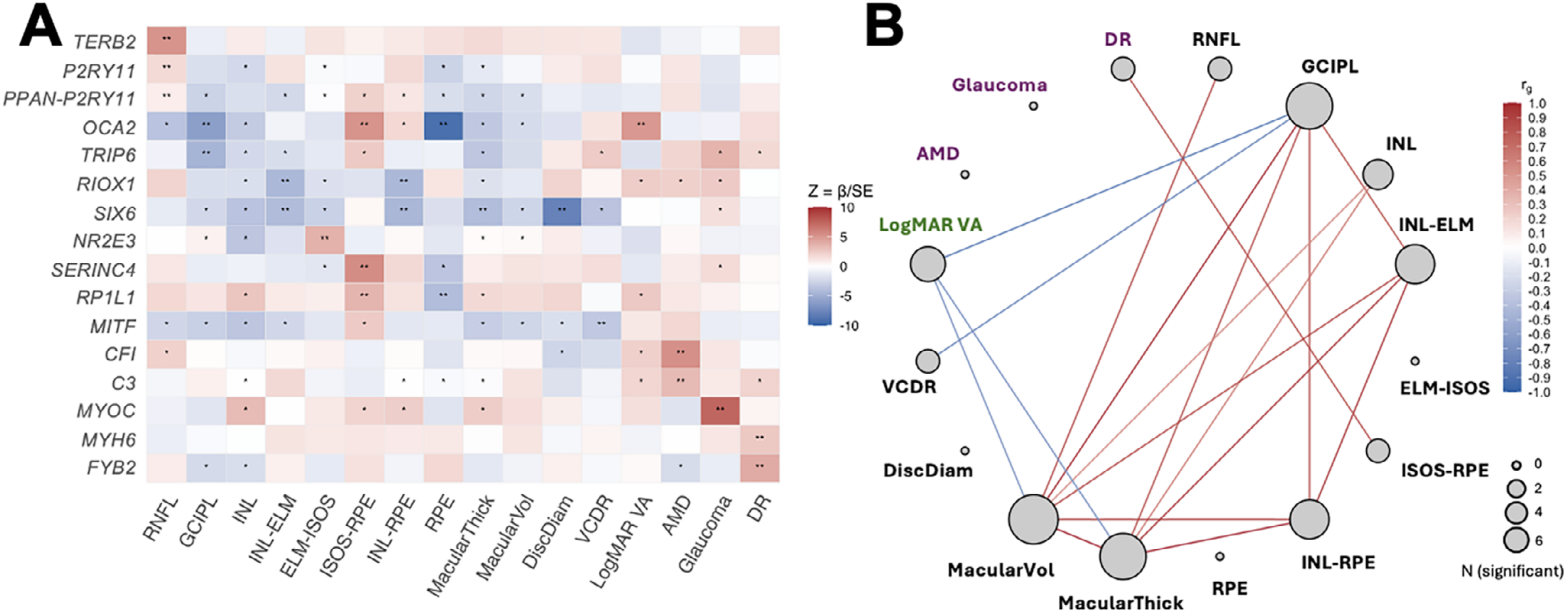
Cross‐phenotype pleiotropy of retinal‐associated genes. (A) Heatmap illustrating pleiotropic effects of the rare‐variant‐associated genes identified in this study across retinal phenotypes. The color scale represents the direction and magnitude of association Z‐scores (β/SE). *P* values are derived from gene‐based rare‐variant association analyses using SAIGE‐GENE+. Multiple testing correction was performed using the Benjamini‐Hochberg false‐discovery rate (FDR) at 0.05. Two asterisks indicate FDR‐significant associations (FDR < 0.05), and one asterisk indicates nominal significance (*p* < 0.05). (B) Chord diagram illustrating pairwise genetic correlations among the 16 retinal phenotypes, including optical coherence tomography (OCT) structural traits, visual acuity (logMAR VA), age‐related macular degeneration (AMD), glaucoma, and diabetic retinopathy (DR). Edge color denotes the genetic correlation coefficient (rg), with red indicating positive and blue indicating negative correlations. Node size is proportional to the number of significant rare‐variant gene associations for each phenotype.

To characterize cross‐phenotype effects, we summarized gene‐trait associations across retinal structure, visual function, and disease outcomes (Figure 4A and Table S9). Three genes, *CFI*, *C3*, and *RIOX1* exhibited pan‐phenotypic pleiotropy with significant signals across all three domains, demonstrating shared pathways linking retinal morphology, visual acuity, and disease susceptibility. Several genes showed two‐domain pleiotropy, including *OCA2* with structural layers and visual acuity, *RP1L1* with photoreceptor and RPE‐related layers and visual function, *MYOC* with glaucoma and structural measures, and *FYB2* with DR and retinal structure. Collectively, the convergence of signals across multiple independent domains provides multi‐level evidence supporting the robustness of these associations, highlighting broader structure‐function‐disease connections and motivating systematic assessment of rare‐variant genetic overlap.

To further quantify genetic correlations (rg) across the 16 traits, we first estimated burden heritability (h^2^) for all 16 retinal phenotypes using burden heritability regression (BHR) across three functional categories (LoF+missense, LoF only, missense). (Figure S3 and Table S10). Missense and LoF+missense variants explained the majority of heritability, whereas LoF only variants contributed minimally. OCT‐derived structural traits showed the highest rare‐variant heritability (median ≈ 2%), followed by visual acuity (∼1%). Disease endpoints (AMD, glaucoma, DR) demonstrated low heritability (<0.5%), indicating that rare coding variants primarily influence retinal morphology and visual function rather than disease susceptibility. Using these heritability estimates, we quantified genetic correlations (rg) across the 16 traits using BHR (Table S11). Figure 4B displays these correlations in a chord diagram, which summarizes the significant correlations across traits. Structural phenotypes such as GCIPL, macular thickness, macular volume, and INL‐related layers formed central hubs with the largest node sizes and numerous positive correlations, indicating that OCT‐derived morphological traits share substantial rare‐variant genetic architecture. LogMAR VA showed strong negative correlations with structural traits, particularly GCIPL and macular measures, supporting structure‐function coupling. In contrast, disease phenotypes (AMD, glaucoma, DR) showed limited genetic correlations with other retinal traits, probably due to their multifactorial and environmentally influenced nature [22, 23].

### 2.5 Tissue and Cell‐Type Localization of Rare‐Variant Genes and Functional Validation of FYB2 in Diabetic Retinopathy

**FIGURE 5:**
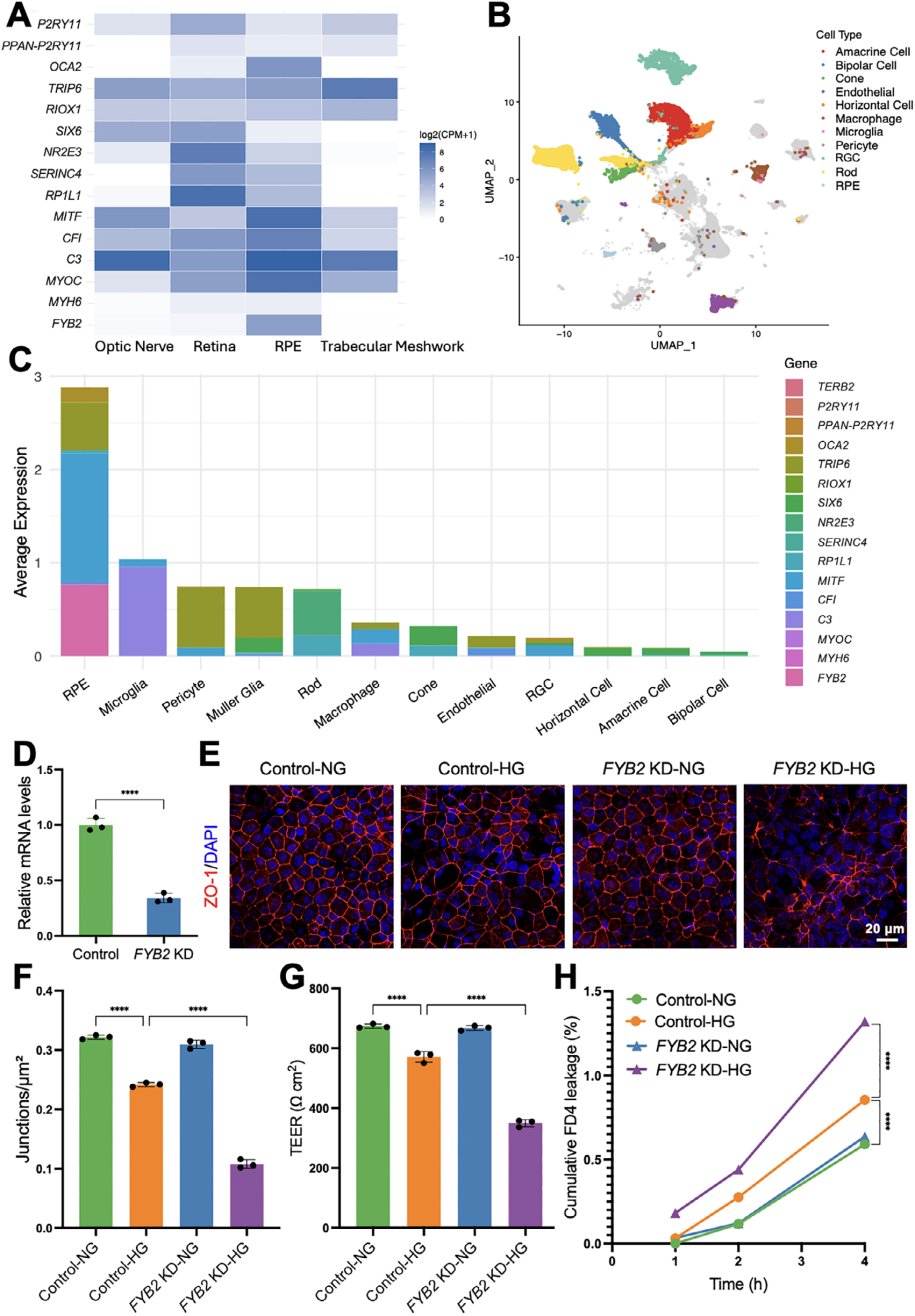
Retinal tissue and cell‐type localization and functional validation of *FYB2* in diabetic retinopathy. (A) Heatmap showing bulk RNA‐seq tissue expression of rare‐variant‐associated genes across optic nerve, retina, retinal pigment epithelium (RPE), and trabecular meshwork, derived from the EyeIntegration transcriptome data. Expression values are shown as log2(CPM + 1). (B) Uniform Manifold Approximation and Projection (UMAP) visualization of single‐cell transcriptomic data from the scEiaD integrated ocular atlas, highlighting 12 major retinal and ocular cell types. (C) Bar plot showing average expression of significant genes across cell types. Expression levels were aggregated by cell category, with RPE, microglia, pericytes, and Müller glia exhibiting the highest expression levels. (D) Quantitative PCR analysis confirming efficient knockdown of *FYB2* (*FYB2* KD) in induced retinal pigment epithelium (iRPE) cells. Relative *FYB2* mRNA levels are shown in control and *FYB2* knockdown groups. Data are presented as mean ± SEM. Statistical significance was determined using an unpaired two‐tailed Student's *t* test. (E) Representative immunofluorescence images of ZO‐1 staining in iRPE monolayers under normal glucose (NG) and high glucose (HG) conditions, with or without *FYB2* KD. Nuclei were counterstained with DAPI. Scale bar, 20 µm. (F) Quantification of junction density in iRPE monolayers under the indicated conditions, showing reduced junction integrity after *FYB2* KD under HG conditions. Data are presented as mean ± SEM. Statistical significance was determined using one‐way ANOVA followed by Tukey's multiple‐comparisons test. (G) Transepithelial electrical resistance (TEER) measurements of iRPE monolayers under the indicated conditions, showing impaired barrier function in *FYB2* KD cells exposed to high glucose. Data are presented as mean ± SEM. Statistical significance was determined using one‐way ANOVA followed by Tukey's multiple‐comparisons test. (H) Cumulative FD4 leakage assay showing increased permeability of iRPE monolayers after *FYB2* KD under HG conditions. Statistical significance was determined using one‐way ANOVA followed by Tukey's multiple‐comparisons test at the 4 h endpoint. ****p < 0.0001.

EyeIntegration data, which reflects tissue‐specific localization patterns, showed that most rare‐variant genes were highly expressed in the retina and RPE, with additional expression in the optic nerve and trabecular meshwork (Figure 5A). *TERB2* was not detected in Gene 2023, the gene‐level expression dataset. *C3* and *CFI* were broadly expressed, with particularly high levels in the RPE. *NR2E3* and *RP1L1* were predominantly enriched in the retina, reflecting their established functions in photoreceptor differentiation [24] and maintenance [25]. *MYOC* was expressed in the retina, RPE, and trabecular meshwork, in line with its role in ocular hypertension and glaucoma [26].

Single‐cell profiling (scEiaD) further resolved the localization of these genes across 12 retinal cell types (Figure 5B,C). Overall expression was highest in the RPE, followed by microglia, pericytes, and Müller glia, with moderate expression in photoreceptors. The enrichment of rare‐variant genes in RPE suggested the participation of these genes in retinal barrier homeostasis and inflammatory regulation.

Among the DR‐associated genes, *FYB2* was selected for downstream functional analysis because DR was the only major disease phenotype in our study that yielded novel rare‐variant associations, and *FYB2* showed expression in RPE‐related contexts, whereas *MYH6* lacked clear enrichment in ocular cell types. To explore its potential role in DR‐relevant retinal pathology, we therefore performed functional validation of *FYB2* in a high glucose iRPE model.

Efficient knockdown of *FYB2* in induced retinal pigment epithelium (iRPE) cells was confirmed by qPCR (Figure 5D). Under normal glucose conditions, *FYB2* silencing caused only minimal disruption of junctional organization. In contrast, under high glucose conditions, *FYB2* knockdown markedly exacerbated barrier dysfunction, as evidenced by increased ZO‐1 discontinuity, reduced junction density, decreased transepithelial electrical resistance (TEER), and increased 4‐kDa fluorescein isothiocyanate‐dextran (FD4) leakage (Figure 5E–H). These findings support a role for *FYB2* in maintaining RPE barrier integrity under diabetic stress and provide experimental support for the biological relevance of the DR‐associated rare‐variant signal.

### 2.6 Biological and Functional Characterization of Rare‐Variant Genes

GO analysis revealed enrichment in retinal developmental programs (eye and visual development, photoreceptor and neuronal differentiation), immune‐complement pathways, pigmentary processes, actin‐myosin cytoskeletal structures, and neurotransmitter receptor signaling (Figure S4A and Table S12). These results suggest that rare variants act through coordinated developmental, immune, pigmentary, cytoskeletal, and neuro‐signaling mechanisms.

We further used AlphaMissense to assess predicted pathogenicity of rare variants (Figure S4B and Table S13). We identified 43 likely pathogenic variants, with high‐confidence ones defined by AlphaMissense scores >0.8. Notably, variants in developmental and pigmentary regulators (*SIX6* and *OCA2*), the photoreceptor transcription factor *NR2E3*, and the cytoskeletal adaptor *TRIP6* showed the highest pathogenicity scores.

To evaluate downstream protein consequences of rare variants, we conducted proteome‐wide association analyses (PWAS) comparing plasma protein levels between variant carriers and non‐carriers. Three signals reached FDR significance (Table S14). Carriers of *MITF* variants exhibited elevated KIT protein levels (β = 0.11, *p* = 2.9 × 10^−12^). Although KIT has not been associated with optic nerve traits in prior publications, this protein‐level signature is mechanistically consistent with the canonical *MITF*‐KIT melanocyte regulatory pathway [27]. Combined with our observed *MITF*‐vertical cup to disc ratio (VCDR) association in the WES analysis, this warrants further investigation of pigment‐related mechanisms in optic nerve morphology. *MYH6* variant carriers exhibited increased protein levels of SCARB1 (β = 0.51, *p* = 6.8 × 10^−8^) and TIMP2 (β = 0.23, *p* = 7.7 × 10^−8^). Given that *MYH6* emerged as a novel DR‐associated gene in our WES analysis, the PWAS signal for TIMP2 provides mechanistic support, as TIMP2 levels have been reported to be associated with the severity of DR [28].

We next integrated expression and proteomic quantitative trait locus (eQTL and pQTL) data using summary‐data‐based Mendelian randomization (SMR) to evaluate putative causal effects of gene expression and protein abundance on disease risk. Using whole blood eQTL datasets and plasma pQTL resources (Methods), we identified *CFI* for AMD and *TRIP6* for glaucoma as putative causal candidates supported by SMR and HEIDI results (Table S15). Notably, the causal implication of *TRIP6* in glaucoma has not been previously reported, highlighting a potential novel disease‐related mechanism.

### 2.7 Clinical Relevance of the Identified Rare‐Variant Genes in Retinal Disorders

To assess clinical relevance, we cross‐referenced significant genes with ClinVar and OMIM annotations (Table S16). *CFI* and *C3* were classified as pathogenic or likely pathogenic for AMD [29], and *MYOC* was pathogenic for open‐angle glaucoma [26], fully concordant with our gene‐based associations. Several genes carried pathogenic annotations for inherited eye diseases beyond our phenotypes, highlighting pleiotropy. *RP1L1* and *NR2E3* were linked to retinitis pigmentosa [25], occult macular dystrophy [30], and enhanced S‐cone syndrome [31], while *SIX6* was associated with congenital optic disc and macular anomalies [32]. In addition, *OCA2* and *MITF* were identified to be pathogenic for pigmentation and syndromic disorders [33]. Several other genes (*TRIP6*, *RIOX1*, *SERINC4*, *FYB2*, *TERB2*, *P2RY11*, *PPAN‐P2RY11*, and *MYH6*) showed limited or non‐ocular clinical annotations, suggesting potential novel retinal roles uncovered only through population‐scale exome analysis.

**FIGURE 6:**
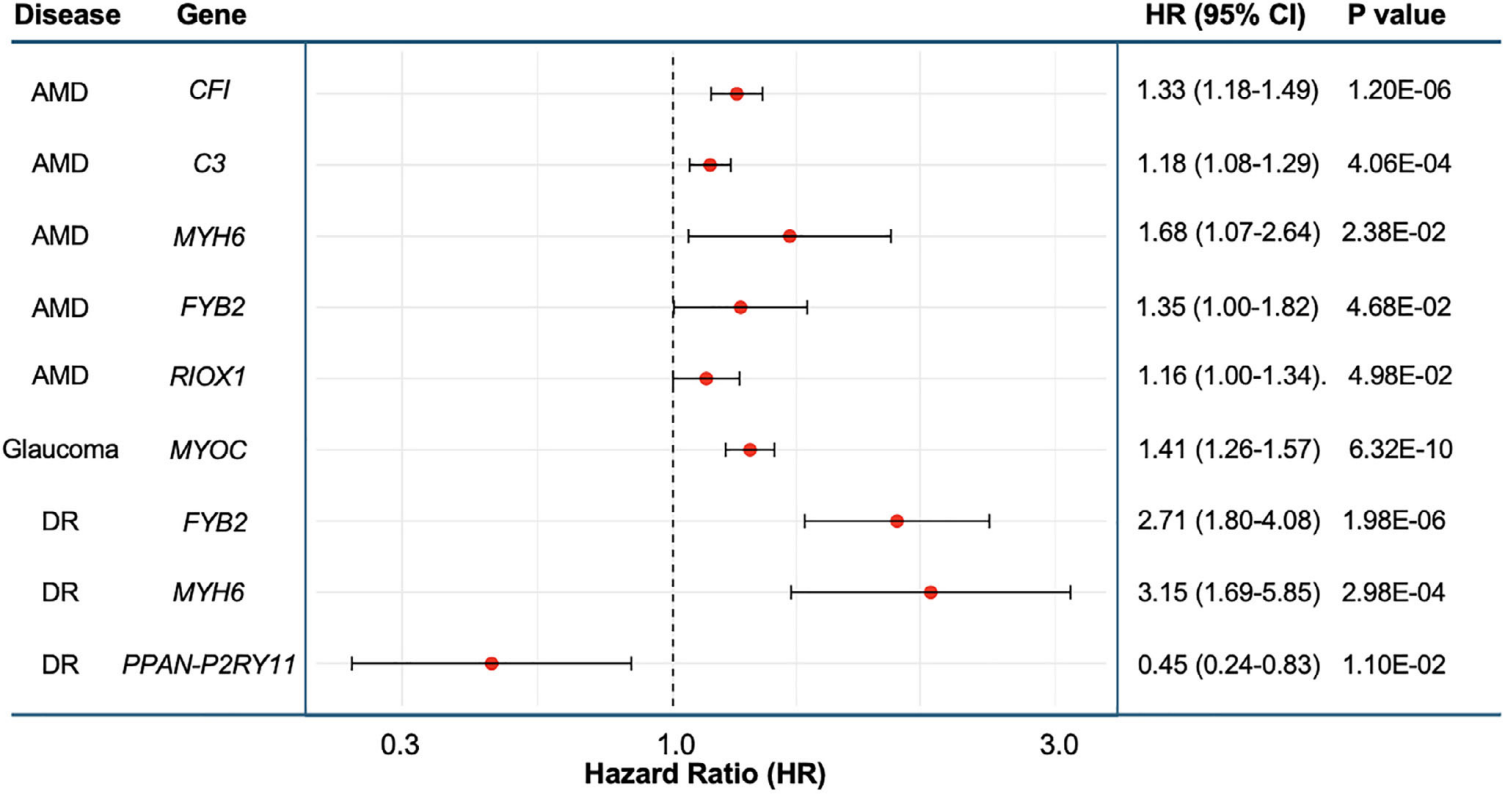
Clinical and functional relevance of the identified rare‐variant genes. Forest plot showing hazard ratios (HRs) with 95% confidence intervals (CIs) for associations between carriers of rare deleterious variants in significant genes and the risk of age‐related macular degeneration (AMD), glaucoma, and diabetic retinopathy (DR). Cox proportional hazards models were fitted using age as the time scale, adjusted for sex and principal components of ancestry. Red dots represent HR point estimates, and horizontal bars represent 95% CIs. The vertical dashed line indicates HR = 1. Only gene‐disease associations with *p* < 0.05 in Cox regression are displayed.

We next evaluated whether rare‐variant carriers exhibited increased longitudinal risk of AMD, glaucoma, and DR using Cox regression. At the aggregate level, after adjustment for sex and population structure, rare variant carrier status was associated with a modest but statistically significant increased risk of age‐related macular degeneration (HR = 1.08, 95% CI 1.04‐1.13, p = 3.91 × 10^−4^) and glaucoma (HR = 1.05, 95% CI 1.01‐1.10, p = 9.26 × 10^−3^), but not diabetic retinopathy. At the gene‐specific level (Figure 6 and Table S18), well‐established disease genes were confirmed: *CFI* (HR = 1.33, 95% CI, 1.18‐1.49; *p* = 1.2 × 10^−6^) and *C3* (HR = 1.18, 95% CI, 1.08‐1.29; *p* = 4.1 × 10^−4^) for AMD, and *MYOC* (HR = 1.41, 95% CI, 1.26‐1.57; *p* = 6.3 × 10^−10^) for glaucoma. Novel loci included *MYH6*, *FYB2* and *RIOX1* for AMD; *FYB2* and *MYH6* for DR; and notably, *PPAN*‐*P2RY11* carriers showed reduced DR risk (HR = 0.45, 95% CI, 0.24‐0.83; *p* = 0.011). Together, these analyses validate established clinical genes and highlight additional rare‐variant candidates contributing to long‐term retinal disease risk.

### 2.8 Pleiotropic Extensions to Brain and Systemic Traits

**FIGURE 7:**
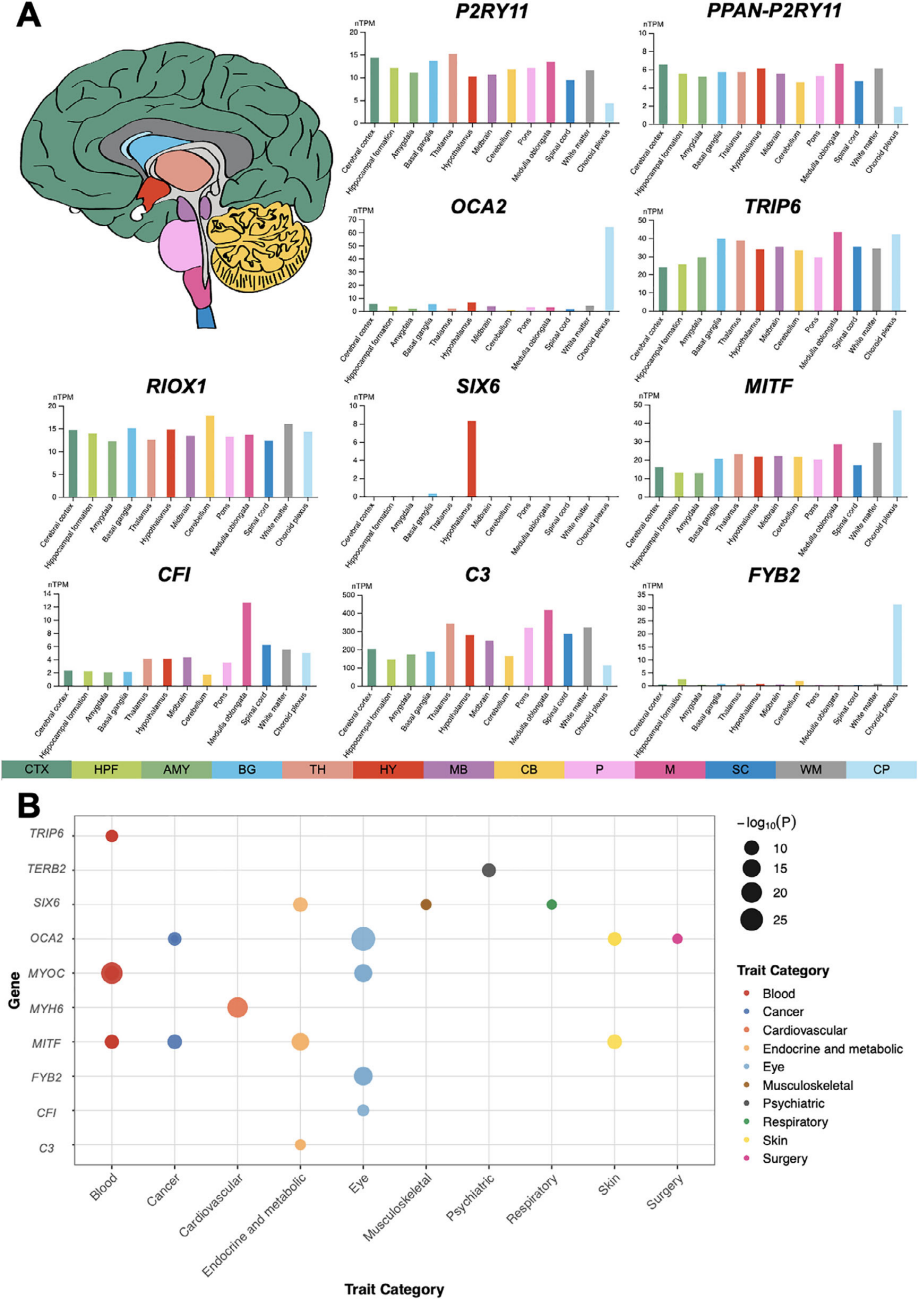
Brain expression profiles of significant retinal‐associated rare‐variant genes. (A) Bar plots showing normalized transcript expression (nTPM) of significant genes across major human brain regions based on the Human Protein Atlas Brain Atlas. Each subplot corresponds to one gene, and bars represent mean expression levels across cortical and subcortical structures, including the cerebral cortex (CTX), hippocampus (HPF), amygdala (AMY), basal ganglia (BG), thalamus (TH), hypothalamus (HY), midbrain (MB), cerebellum (CB), pons (P), medulla (M), spinal cord (SC), white matter (WM), and choroid plexus (CP). The schematic on the left depicts the anatomical localization of these regions. (B) System‐level pleiotropy of significant genes across trait categories. Each bubble represents a significant gene‐trait association (*p* < 5 × 10^−8^) from the Genebass and All of Us datasets. Bubble size is proportional to ‐log_10_(P) and color indicates the corresponding trait category.

As the retina is a specialized extension of the CNS, we profiled gene expression across brain regions to assess eye‐brain convergence. Multiple rare‐variant genes showed distinct CNS expression, consistent with an eye‐brain axis (Figure 7A). Developmental regulator *SIX6* displayed pronounced enrichment in the hypothalamus, consistent with neurodevelopmental roles extending beyond the retina. The immune adaptor *FYB2* showed the strongest enrichment in the choroid plexus (CP), which is an immune interface analogous to the blood‐retina barrier, and *OCA2* also exhibited its highest expression in this region. These patterns reflect distinct CNS‐expression signatures among retinal‐associated genes.

Systemic pleiotropy analyses through phenome‐wide association scans (PheWAS) using Genebass and All of Us revealed that many retinal coding‐variant genes extend their effects beyond ocular traits into broader physiological domains (Figure 7B and Table S19). Pigmentation‐related regulators *OCA2* and *MITF* showed the widest cross‐system footprint, spanning dermatologic, hematologic, and metabolic traits as well as cancer phenotypes, consistent with pleiotropic roles of melanocytic and metabolic signaling pathways. Developmental gene *SIX6* demonstrated associations with endocrine‐metabolic traits and musculoskeletal and respiratory phenotypes, suggesting coordinated developmental influences across multiple organ systems. Immune‐modulatory gene *C3* displayed associations with endocrine and metabolic traits, aligning with its roles in complement signaling and immune regulation. Collectively, these data demonstrate systemic pleiotropy across pigmentation, immune, metabolic, and cardiopulmonary traits.

## 3 Discussion

In this large‐scale WES analysis integrating retinal structure, visual function, and major blinding retinal diseases, we define a comprehensive landscape of protein‐coding variation in the human retina. We identified 16 genes across 22 gene‐based rare‐variant associations, of which 12 were novel. Ten of these novel genes were further externally replicated in the All of Us cohort through associations with blindness and low vision and with disorders of optic nerve and visual pathways, supporting their robustness and biological relevance. Complementing these results, common‐variant analyses identified an additional 26 novel associations. We further uncovered retinal structure‐function‐disease pleiotropy, demonstrated biological and clinical relevance, and provided preliminary evidence for extensions into brain and systemic health.

Large‐scale WES refines the retinal genetic map by capturing rare protein‐coding variants missed by genotyping arrays, leading to 12 novel gene discoveries and demonstrating the value of WES for retinal genetic discovery. Among them, *MYH6* and *FYB2* emerged as novel risk genes for DR. Both genes were externally replicated in the All of Us cohort, supporting their robustness across ancestries, and our Cox analyses further validated their clinical relevance to DR and AMD. For *MYH6*, carriers exhibited elevated circulating levels of TIMP2 in our PWAS. TIMP2, an endogenous inhibitor of matrix metalloproteinases (MMPs), plays a key role in extracellular matrix remodeling [34]. Importantly, its expression increases with DR severity, consistent with progressive vascular and fibrotic changes in advanced DR [28]. These findings provide a biologically plausible link between *MYH6* and microvascular pathology in DR. *FYB2* encodes a key immune adaptor in T‐cell receptor‐mediated activation of signaling pathways and T‐cell activation [35]. In our experimental validation, *FYB2* knockdown exacerbated barrier dysfunction in high‐glucose iRPE cells, supporting a potential role in maintaining RPE barrier integrity under diabetic stress. Although inflammatory pathways are fundamental to DR, the precise mechanisms linking *FYB2* to retinal barrier dysfunction and DR pathogenesis remain to be clarified. In addition, *TRIP6* was found to be a novel rare‐variant gene for GCIPL, and its pathogenicity was predicted to be deleterious with a high confidence score by AlphaMissense. *TRIP6* is known to be an action and migration‐modulating factor [36], and our GO enrichment results highlight its involvement in contractile actin cytoskeletal structures. Our SMR analysis suggested a potential causal effect on glaucoma, consistent with established mechanisms of cytoskeletal dysregulation in glaucoma [37], although its specific role requires further investigation. In addition, WES also provided added value for common coding variation with 26 novel associations not previously reported in the GWAS Catalog, illustrating that population‐scale WES not only refines rare‐variant architecture but also enhances resolution within the common coding genome.

For genes that were previously established, such as C3 and CFI in AMD and MYOC in glaucoma, our study revealed additional rare‐variant contributions, reinforcing their gene‐level involvement beyond signals driven predominantly by common variants. Genes such as OCA2 and SIX6 emerged as extended associations with high pathogenicity scores by AlphaMissense. WES identified gene‐level associations between *OCA2* and visual acuity as well as multiple retinal layers including GCIPL, ISOS‐RPE, and RPE, extending its known roles in pigmentation biology [38] and visual function in patients with albinism [21]. SIX6 was found to be associated with disc diameter and multiple retinal layers including macular thickness, INL‐RPE and INL‐ELM, in alignment with its broader involvement in retinal and optic‐nerve development [39]. Together, these findings expand the spectrum of coding variation influencing retinal structure, function, and disease, and underscore the value of WES as a complementary approach to GWAS for dissecting mechanistic pathways and enhancing translational insight in retinal genetics.

We further observed broad cross‐phenotype pleiotropy within the retina, suggesting that coding variation may exert coordinated effects across morphology, visual performance, and clinical disease. Three genes—*CFI*, *C3*, and *RIOX1*—exhibited three‐dimensional structure‐function‐disease pleiotropy, positioning them as central regulatory nodes that couple retinal architecture with downstream functional and pathological consequences. As the complement system is critical in maintaining retinal integrity and visual function [40], coding variation in *CFI* and *C3* may simultaneously affect retinal structure, functional performance, and disease risk. *RIOX1* encodes a protein involved in ribosome biogenesis and replication [41] and emerged as a novel risk gene for both INL‐ELM and INL‐RPE thickness in our ExWAS, suggesting a potential influence on photoreceptor‐associated layers. *RIOX1* was also found to be associated with visual acuity as well as AMD and glaucoma in our pleiotropy analyses, implicating *RIOX1* as a previously unrecognized regulator operating along a coordinated structure‐function‐disease axis in the retina.

Retinal rare‐variant genes also showed extensions beyond ocular phenotypes, providing insight into broader brain and systemic pathways. Cross‐tissue expression profiling demonstrated that several rare‐variant genes exhibit selective enrichment across distinct brain regions, delineating a genetically anchored axis of neurodevelopmental and neuroimmune regulation. SIX6 showed marked enrichment in the hypothalamus, consistent with its established developmental roles [42] and suggesting shared genetic programs that couple retinal and central neurodevelopment. *FYB2* and *OCA2* were strongly enriched in the choroid plexus—an immune interface analogous to the blood‐retina barrier—highlighting shared neuroimmune architecture between ocular and central nervous tissues. Together, these CNS signatures point to conserved pathways governing neurodevelopment, immune surveillance, and barrier integrity across eye and brain. Beyond the CNS, PheWAS revealed that these genes also influence systemic traits across pigmentation, inflammation, metabolism, and cardiopulmonary physiology. Pigmentation regulators *OCA2* and *MITF* exhibited the broadest cross‐system footprint, spanning dermatologic, hematologic, metabolic, and neoplastic phenotypes—consistent with the shared melanocytic and metabolic origins of these traits. Developmental regulator *SIX6* was associated with cardiopulmonary and anthropometric traits, suggesting broader developmental scaling effects beyond the visual system. Complement gene *C3* was linked to immune and metabolic traits, reflecting its important role in systemic inflammation. Collectively, these findings position the retina not merely as a specialized sensory organ but as a window into shared neurodevelopmental, immune, and metabolic biology across systems.

The strengths of this work lie in coupling large‐scale WES with multidimensional retinal phenotyping, which uncovers a coding‐variant framework that shapes retinal health and disease. By bringing together structural, functional, and disease endpoints, we outline a three‐dimensional pleiotropic architecture that links coding variation to measurable retinal changes and disease risk. Furthermore, the incorporation of cross‐tissue expression profiling and phenome‐wide analyses offers broader insight into their potential relevance in brain and systemic biology, positioning retinal coding‐variant genes within shared neurodevelopmental, neuroimmune, and metabolic pathways across organ systems. These multi‐layer mechanistic and clinical analyses together provide a translational framework connecting genotype to both ocular and systemic health. Several limitations should be acknowledged. First, despite the large sample size, analyses were restricted to individuals of European ancestry to minimize population stratification; replication in multi‐ancestry cohorts will be essential to establish global generalizability. Second, WES captures only protein‐coding variation, leaving noncoding regulatory variants—likely contributors to retinal gene expression regulation—unexplored. Future work should incorporate whole‐genome sequencing in diverse populations to resolve regulatory contributors and refine fine‐mapping across ancestries. Third, retinal layer thickness measurements were derived from UK Biobank OCT images using the widely adopted Topcon Advanced Boundary Segmentation algorithm without manual verification of segmentation outputs. Future studies incorporating expert manual grading or deep learning‐based refinement of retinal layer segmentation may further improve measurement precision. Fourth, rare‐variant association analyses have inherent limitations and should be interpreted with caution, as low carrier counts can limit statistical power and replication [43], ancestry‐specific variant spectra may affect generalizability [44], and analytical choices in variant aggregation and statistical testing may influence interpretation [45]. Lastly, although our experimental validation provided preliminary support for *FYB2* in diabetic stress‐related RPE barrier dysfunction, more extensive functional studies will be necessary to dissect the complement, pigmentary, and neuroimmune pathways implicated here. Collectively, these efforts will advance the mechanistic interpretation of coding variation and accelerate translation toward predictive and therapeutic applications.

In summary, our study provides a large‐scale WES‐based genetic map of the human retina, identifying novel coding‐variant associations and refining the genetic architecture of retinal structure, visual function, and major blinding diseases. We further delineate coordinated structure‐function‐disease pleiotropy within the retina and offer preliminary evidence that these coding‐variant associations may extend into brain and systemic traits. Together, these findings outline a multi‐layer genetic framework that advances understanding of retinal biology and informs future mechanistic and translational research.

## 4 Materials and Methods

### 4.1 Study Population and Phenotype Definition

The UKB is a large prospective population‐based cohort of over 500,000 adults aged 40–69 years recruited across the United Kingdom, with genetic, phenotypic, and health‐related data collected at baseline assessment and during follow‐up.

The phenotype definitions were consistent with previous UKB studies [10, 11]. Retinal optical coherence tomography (OCT) imaging was performed using Topcon instruments, including the Topcon 3D OCT‐1000 Mk2 and Topcon Triton. OCT‐derived structural parameters were generated using the Topcon Advanced Boundary Segmentation (TABS) software [46] and obtained from the UK Biobank Data Showcase under Category 100079 and Category 1081. In total, 88 OCT‐derived parameters are provided, including both global summary measures and subfield‐specific metrics based on the Early Treatment Diabetic Retinopathy Study (ETDRS) grid. To ensure interpretability and minimize redundancy, we focused on 12 global structural traits summarizing retinal morphology at the whole‐eye level. These comprised average layer thicknesses (RNFL, GCIPL, INL, INL‐ELM, ELM‐ISOS, ISOS‐RPE, INL‐RPE, RPE), macular parameters (overall macular thickness and macular volume), and optic nerve head measurements (disc diameter and VCDR). We averaged left and right eye measurements when available to derive a single trait per individual.

Visual function was assessed using logMAR visual acuity (UKB fields 5208 and 5201 for the left and right eyes, respectively). When data were available for both eyes, mean values were calculated.

Three vision‐threatening retinal diseases (AMD, glaucoma, and DR) were defined using multiple sources: (i) hospital inpatient records with primary and secondary diagnoses from ICD‐10 and ICD‐9 codes; (ii) the touchscreen self‐report of “eye problems/disorders” (data field 20002; responses including “macular degeneration” or “glaucoma”); and (iii) the self‐reported non‐cancer illness dataset (data field 6148; responses including “macular degeneration” or “glaucoma”). Control individuals were defined as participants without any diagnosis or self‐report of ocular diseases. Detailed coding schemes and sample sizes for each trait are summarized in Table S2.

### 4.2 Exome Sequencing and Quality Control

Whole‐exome sequencing (WES) was performed by the Regeneron Genetics Center (RGC) using the IDT xGen Exome Research Panel v1.0 on the Illumina NovaSeq 6000 platform, achieving >95% coverage at 20× depth [47]. We used the OQFE WES pVCF files aligned to GRCh38 provided by the UK Biobank (data field 23148).

In addition to the centralized quality control conducted by the UKB team, we applied further genotype‐level, variant‐level, and sample‐level quality control (QC) steps following established protocols to ensure data integrity and reliability for downstream analyses [14, 15]. Briefly, genotype‐level QC was first conducted by decomposing multi‐allelic sites into bi‐allelic variants and setting low‐quality or extreme‐depth genotype calls to missing. Second, variant‐level QC excluded variants with call rate <90%, marked deviations from Hardy‐Weinberg equilibrium, or presence in low‐complexity regions. Third, sample‐level QC removed individuals who had withdrawn consent, duplicates, sex discordance, or extreme outlier values in Ti/Tv, Het/Hom, SNV/indel ratios, or singleton counts.

To minimize population stratification, analyses were limited to unrelated Caucasian participants (field 22006). Related individuals were removed at a kinship threshold of 0.0884 to ensure an unrelated sample, and within‐ancestry principal components (PCs) were computed from high‐quality variants.

### 4.3 Variant Annotation and Functional Classification

All variants were uniformly annotated using SnpEff v5.1 [48] with Ensembl Build 38 gene models and ANNOVAR with the RefGene annotation database [49]. Predicted loss‐of‐function (pLoF) variants were defined as frameshift insertions/deletions, splice donor/acceptor disruptions, stop‐gain, stop‐loss, or start‐loss mutations. Predicted deleterious missense (pMis) variants were defined as missense substitutions consistently classified as damaging by at least five independent in silico predictors (SIFT [50], LRT [51], PolyPhen‐2 HDIV and PolyPhen‐2 HVAR [52], and MutationTaster [53]). Variant consequence categories were harmonized across SnpEff and ANNOVAR outputs to ensure consistent functional classification. Allele frequencies were estimated within the UKB WES dataset and compared with gnomAD and were subsequently used to distinguish rare (MAF <1%) from common (MAF ≥1%) variants for downstream analyses.

### 4.4 Exome‐Wide Gene‐Based Association Analyses (ExWAS)

Gene‐based association tests were performed using SAIGE‐GENE+ (v1.1.6.2) [54] to evaluate the aggregate effects of rare coding variants. Eight variant‐collapsing schemes were applied, combining four MAF thresholds (<1 × 10^−5^, <1 × 10^−4^, <0.001, and <0.01) with two functional masks—predicted loss‐of‐function (LoF) only, and LoF plus deleterious missense (Mis) variants. Models were adjusted for age, sex, and the top 10 genetic principal components (PCs). A sparse genetic relationship matrix (GRM) constructed from high‐quality variants (pairwise relatedness <0.05) was included as a random effect. Gene‐level statistics were computed using the SKAT, burden, and SKAT‐O tests, with *p* values from the SKAT‐O test reported as the primary results. Exome‐wide significance was defined as *p* < 2.5 × 10^−6^. Global FDR correction was performed across all gene‐phenotype‐mask combinations, and associations with an overall FDR < 0.05 were considered statistically significant.

### 4.5 Leave‐One‐Variant‐Out (LOVO) and Conditional Analyses

LOVO analyses were conducted to determine whether the observed gene‐level associations were driven by single influential variants. A variant was considered influential if its exclusion rendered the gene non‐significant (*p* > 0.001), suggesting that the signal was dominated by that variant.

Conditional analyses were then performed to evaluate independence from nearby common variants. For each significant gene, imputed variants with MAF ≥ 1% located within ±500 kb (UKB field 22828) were extracted and pruned using linkage disequilibrium (LD) clumping (clump‐p1 = 1 × 10^−5^, clump‐r^2^ = 0.01) implemented in PLINK v2.0 to identify independent index variants. These lead SNPs were included as covariates in rerun collapsing tests to assess the independence of the rare variant signal from surrounding common variant loci.

### 4.6 Cross‐Reference and External Replication

To evaluate the novelty and robustness of rare variant gene‐based associations identified in the ExWAS, we cross‐referenced significant gene‐trait pairs against several publicly available resources: (i) Genebass (https://genebass.org), which reports rare‐variant gene‐based associations from the UKB exome dataset, (ii) the GWAS Catalog, a curated repository of genome‐wide association study findings, and (iii) PubMed. Associations were classified as: previously reported, extended (related traits or SNP‐level evidence), or novel.

For external replication, we further queried the All of Us Research Program database (https://allbyall.researchallofus.org), which provides multi‐ancestry gene‐level rare variant association statistics, serving as an independent validation dataset to evaluate the consistency of the identified associations across populations.

### 4.7 Single‐Variant Analysis for Common Variants

Single‐variant association tests for common variants (MAF ≥ 1%) were conducted using linear mixed model regression implemented in PLINK v2.0 [55]. Analyses were adjusted for age, sex, and the top ten principal components (PCs). The significance threshold was established at the conventional threshold for GWAS (*p* < 5 × 10^−8^). Lead SNPs were identified through linkage disequilibrium (LD)‐based clumping (±1 Mb, r^2^ ≤ 0.1). Novel association signals were defined as variants more than 500 kb away from previously reported variants in the GWAS Catalog (https://www.ebi.ac.uk/gwas/).

### 4.8 Tissue‐Type Enrichment Analysis

Tissue‐specific expression of exome‐wide significant genes was examined using the EyeIntegration portal (https://eyeintegration.nei.nih.gov/), a harmonized transcriptome atlas of human ocular and systemic tissues. Normalized expression data were used to identify preferential enrichment in retina, RPE, optic nerve, or trabecular meshwork relative to non‐ocular tissues based on the annotation framework provided in EyeIntegration.

### 4.9 Single‐Cell Expression Profiling

Single‐cell RNA‐sequencing data were obtained from the scEiaD integrated atlas (https://plae.nei.nih.gov/), which aggregates large‐scale datasets across multiple retinal and ocular studies. The atlas provides uniformly processed expression matrices, metadata, UMAP embeddings, and curated annotations for 12 major retinal cell populations.

To quantify cell‐type‐specific enrichment, we computed a signature score for each cell, defined as the mean normalized expression of the rare variant‐associated genes identified in this study. Visualization and quantification were performed in R using the ggplot2 and dplyr packages. Mean signature scores were then averaged across annotated cell types to quantify enrichment within specific retinal populations.

### 4.10 Cross‐Phenotype and Heritability Analyses

To assess genetic pleiotropy across retinal traits, two‐sided Fisher's exact tests were used to quantify the overlap of significant gene‐based associations across all phenotype pairs. Associations were evaluated at both the nominal (*p* < 0.05) and exome‐wide (*p* < 2.5 × 10^−6^) significance levels.

We next applied Burden Heritability Regression (BHR) [56] to estimate the contribution of rare coding variants to the heritability of 16 retinal phenotypes. BHR models the relationship between gene‐based association statistics and aggregated burden scores, with the regression slope representing the heritability estimate. Three functional annotation masks were analyzed: (i) loss‐of‐function (LoF) plus deleterious missense variants, (ii) LoF variants only, and (iii) missense variants only. Rare‐variant genetic correlations (r_g_) were estimated for all pairwise phenotype combinations using the bivariate mode of BHR.

### 4.11 Cell Culture and Glucose Treatment

Induced retinal pigment epithelium (iRPE) cells derived from the human embryonic stem cell line ES9 were used in this study. Cells were cultured in Miller medium, which supports the formation of functionally polarized human RPE monolayers [57]. The medium consisted of α‐MEM (M4526, Sigma–Aldrich, USA) supplemented with 5% fetal bovine serum (FBS; A5256701, Gibco, USA), taurine (125 mg; T0625‐10G, Sigma‐Aldrich, USA), N1 supplement (N6530‐5ML, Sigma–Aldrich, USA), non‐essential amino acids (NEAA; 11140050, Thermo Fisher Scientific, USA), GlutaMAX (35050061, Invitrogen, USA), hydrocortisone (HY‐N0583, MedChemExpress, USA), and triiodothyronine (S5726, Selleck, USA). Cells were maintained at 37°C in a humidified atmosphere containing 5% CO2. For glucose treatment, iRPE cells were cultured in normal glucose (NG, 5 mm) or high glucose (HG, 30 mm) medium for 35 days prior to downstream analyses.

### 4.12 Lentivirus Construction and Gene Knockdown

Lentiviruses for stable FYB2 knockdown were commercially packaged by Hanbio Biotechnology (China). An shRNA targeting FYB2 was cloned into the pHBLV‐U6‐MCS‐CMV‐ZsGreen‐PGK‐PURO vector, and the targeting sequence was 5′‐GCAAATATGGATATGTGCTCATTGA‐3′. Control lentiviruses carried the same empty vector backbone expressing ZsGreen and puromycin resistance.

iRPE cells were transduced with lentivirus at a multiplicity of infection (MOI) of 20 in the presence of 4 µg/mL polybrene (C0351, Beyotime Biotechnology, China). Twenty‐four hours later, the medium was replaced, and cells were subjected to selection with 2 µg/mL puromycin (ST551, Beyotime Biotechnology, China) until nontransduced control cells were eliminated. Stable cells were subsequently expanded for downstream experiments.

### 4.13 Quantitative Reverse Transcription PCR (qRT‐PCR)

Total RNA was extracted from iRPE cells using a Total RNA Extraction Kit (DP419, Tiangen Biotech, China), and RNA concentration was measured using a NanoDrop 2000 spectrophotometer (Thermo Fisher Scientific, USA). Complementary DNA (cDNA) was synthesized using PrimeScript RT Master Mix (RR036A, TaKaRa Bio, Japan). Quantitative real‐time PCR was performed using SYBR Premix Ex Taq (RR420A, TaKaRa Bio, Japan) on a ViiA 7 Real‐Time PCR System (Applied Biosystems, USA). The cycling conditions were as follows: an initial denaturation at 95°C for 5 min, followed by 40 cycles of 95°C for 10 s, 60°C for 20 s, and 72°C for 20 s. Relative mRNA expression levels were calculated using the 2^−ΔΔCt^ method with *GAPDH* as the internal control. The primer sequences were as follows: *FYB2*, forward ATTGGAGGCACACAGTCAACT, reverse CTGGGACTCACTACTGGAACA; *GAPDH*, forward GGAGCGAGATCCCTCCAAAAT, reverse GGCTGTTGTCATACTTCTCATGG.

### 4.14 Immunofluorescence Staining and Junction Analysis

Cells were seeded onto Transwell inserts and cultured under the indicated conditions. After treatment, cells were fixed with 4% paraformaldehyde (PFA; G1101, Servicebio, China) for 20 min at room temperature, followed by permeabilization and blocking in 5% bovine serum albumin (BSA; ST023, Beyotime Biotechnology, China) containing 0.03% Triton X‐100 (A110694‐0100, Diamond Biotechnology, China) for 1 h at room temperature. Cells were then incubated with an anti‐ZO‐1 antibody (339100, Thermo Fisher Scientific, USA) overnight at 4°C, followed by incubation with the appropriate fluorophore‐conjugated secondary antibody for 1 h at room temperature in the dark. After each incubation step, cells were washed three times with phosphate‐buffered saline (PBS; C14190500CP, Thermo Fisher Scientific, USA). Nuclei were counterstained with DAPI (D1306, Thermo Fisher Scientific, USA). Images were acquired using a confocal laser‐scanning microscope (Carl Zeiss Meditec, Germany).

Quantitative assessment of RPE barrier integrity was performed on ZO‐1 immunofluorescence images as previously described [58]. Briefly, ZO‐1 images were binarized and skeletonized in ImageJ, and junction density was quantified as the number of junctions per µm^2^.

### 4.15 Transepithelial Electrical Resistance (TEER) Measurement

TEER was measured to evaluate barrier function after iRPE cells formed confluent monolayers on Transwell inserts. TEER values (Ω·cm^2^) were calculated by correcting the measured resistance for the background resistance of blank inserts and normalizing to the surface area of the insert.

### 4.16 4‐kDa Fluorescein Isothiocyanate‐Dextran (FD4) Permeability Assay

After monolayer formation, barrier permeability was evaluated using a FD4 assay. Briefly, 200 µL of 1 mg/mL FD4 (FD4‐100MG, Sigma–Aldrich, USA) was added to the apical chamber, and 500 µL of fresh medium was added to the basolateral chamber. At 1, 2, and 4 h after FD4 loading, 100 µL of medium was collected from the basolateral chamber and immediately replaced with an equal volume of fresh medium. FD4 concentrations in the basolateral samples were determined based on a standard curve. The cumulative percentage of FD4 leakage across the cell monolayer was then calculated with correction for the dilution introduced by serial sampling.

### 4.17 Functional Annotation

To further investigate the biological relevance of the rare variant‐associated genes identified in the exome‐wide analyses, we performed Gene Ontology (GO) enrichment analysis using the clusterProfiler package in R, with significance defined as FDR < 0.05 to identify overrepresented biological processes and molecular functions.

### 4.18 AlphaMissense‐Based Pathogenicity Prediction

The pathogenicity of missense variants was evaluated using the AlphaMissense model (https://alphamissense.hegelab.org/), which provides scores ranging from 0 to 1 representing a calibrated estimate of pathogenicity probability. Variants with scores ≥0.564 were considered likely pathogenic in the AlphaMissense released annotations [59]. Given the bimodal distribution of AlphaMissense scores, pathogenic variants are strongly enriched at the upper end of the score spectrum, whereas the intermediate range (0.2–0.8) represents a heterogeneous region with reduced signal separation and lower calibration reliability [59]. Therefore, we defined variants with scores >0.8 as high‐confidence pathogenic candidates to minimize false‐positive predictions.

### 4.19 Proteomic‐Wide Analysis

We performed a proteome‐wide association analysis to evaluate the relationships between rare variant gene carriers and plasma proteomic profiles using the UKB Olink. Analyses were restricted to participants of European ancestry. Covariates included age, sex, and the first ten genetic principal components. Statistical significance was defined as an FDR‐adjusted *p* < 0.05. This analysis aimed to explore potential downstream proteomic effects of the rare variant genes.

### 4.20 Summary‐Data‐Based Mendelian Randomization (SMR)

We performed summary‐data‐based Mendelian randomization (SMR) analyses using the SMR online platform (https://yanglab.westlake.edu.cn/smr‐portal/) to assess whether gene or protein expression levels of the identified genes exert causal effects on ocular diseases. This method integrates summary‐level QTL and GWAS statistics to test whether a shared genetic variant influences both gene or protein expression and disease risk, thereby inferring potential causal relationships.

Given the limited availability of ocular tissue eQTL data, we used blood‐derived eQTL and pQTL datasets as proxies for systemic genetic regulation. Specifically, eQTLGen (*n* = 31,684, whole blood) and GTEx v8 (*n* = 755, whole blood) were used for transcriptomic QTLs, whereas INTERVAL (n = 3,301), Fenland (*n* = 10,708), and SCALLOP (*n* = 30,931) served as plasma proteomic QTL sources. GWAS summary statistics for AMD, glaucoma, and DR were derived from UKB imputed genotype data, adjusting for age, sex, and the first ten genetic principal components.

The SMR framework uses the top cis‐QTLs (within ±1 Mb of each gene) as instrumental variables to estimate causal effects, with the accompanying HEIDI (Heterogeneity In Dependent Instruments) test distinguishing linkage from pleiotropy. Significant SMR results with non‐significant HEIDI tests were interpreted as evidence of shared causal variants [60]. Results with *p* < 0.05 and non‐significant HEIDI tests (*p* > 0.05) were considered consistent with a shared causal variant.

### 4.21 Cox Proportional Hazards Regression Analyses

We performed Cox proportional hazards regression analyses for each significant gene. Rare coding variants were aggregated according to the optimal MAF thresholds and functional masks identified in the exome‐wide analyses, and individuals carrying at least one qualifying variant were defined as carriers.

Time‐to‐event was defined from the date of birth (UKB field 33) to the earliest occurrence of a first diagnosis of AMD, glaucoma, or DR, death, or the last date of hospital admission data available—whichever occurred first. To account for the delayed entry of participants into the cohort (aged 40–69 years at recruitment), left truncation was applied at the date of baseline assessment to correct for the age distribution at enrollment. Models were adjusted for sex and the top ten genetic PCs. After FDR correction, *p* < 0.05 was identified as significant.

### 4.22 Associations of Identified Genes With Brain Protein Expression

Brain‐specific expression patterns of the rare variant‐associated genes were obtained from the Human Protein Atlas Brain Atlas (https://www.proteinatlas.org), which reports bulk RNA‐seq‐based expression (nTPM) across 13 major brain regions, including the cerebral cortex, hippocampus, thalamus, cerebellum, and spinal cord.

For each gene, the maximum nTPM value across brain regions was used to determine regional expression patterns, and genes showing medium to high expression in neuronal tissues were annotated as brain‐enriched. Representative bar plots and immunohistochemistry images were retrieved from the HPA portal for visualization and interpretation.

### 4.23 Phenome‐Wide Association Study (PheWAS)

To explore the systemic pleiotropic effects of rare variant genes associated with retinal phenotypes, we conducted a PheWAS using two large‐scale gene‐level association resources: Genebass and the All of Us Research Program. The Genebass database provides exome‐based gene‐level burden results for approximately 350,000 UKB participants across more than 4,000 quantitative and ICD‐10‐derived disease traits, whereas the All of Us cohort dataset includes gene‐level rare variant association results across thousands of electronic health record (EHR)‐derived phenotypes in roughly 250,000 participants from multiple ancestries.

For each rare variant gene identified in our UK Biobank WES analysis, all significant gene‐trait associations were extracted from both datasets. Associations were considered statistically significant at a genome‐wide threshold of *p* < 5 × 10^−8^. Trait categories were based on the classification framework described previously [61] to facilitate interpretation of systemic pleiotropy.

### 4.24 Statistical Analysis

Data are presented as mean ± SEM. Statistical significance between two groups was evaluated using an unpaired two‐tailed Student's t test. Comparisons among multiple groups were performed using one‐way ANOVA followed by Tukey's multiple‐comparisons test. For the FD4 permeability assay, statistical analysis was performed on the 4 h endpoint using one‐way ANOVA followed by Tukey's multiple‐comparisons test. A value of *p* < 0.05 was considered statistically significant.

## Author Contributions

All authors had full access to all the data in the study and accept responsibility for the decision to submit for publication. B Lu, XD Sun, JT Yu, and XL Wan conceived, designed, and supervised the study. JQ Li, YJ Ge, JX Du, L Yang, BS Wu, and CY Hang analyzed and interpreted the data. MX Shen, YM Mao, T Zhang, QY Bo, TY Zhao, YT Jiao, YZ Wang, SX Gao, JQ Chen, JR Sun, T Li, HX Jia, Y Dou, and W Cheng contributed to data curation, methodological support, result interpretation, and manuscript review. JQ Li and YJ Ge drafted the manuscript. B Lu, XD Sun, JT Yu, and XL Wan critically revised the manuscript. All authors reviewed and approved the final manuscript.

## Ethics Statement

Ethical approval was granted by the North West Multi‐Centre Research Ethics Committee (06/MRE08/65), and all participants provided written informed consent. Our study was conducted under application 202239 and included 356,982 participants (Table S1) with whole‐exome sequencing and clinical data after quality control (data accessed in May 2025).

## Conflicts of Interest

The authors declare no conflict of interest.

## Supporting information




**Supporting File 1**: advs76582‐sup‐0001‐Supplementary Figures.docx.

**Supporting File 2**: advs76582‐sup‐0002‐Revised supplementary table.xlsx.

## Data Availability

The UKB data (https://biobank.ndph.ox.ac.uk/) used in this study were accessed under application number 202239. All software and R packages used in this study are freely available online: SAIGE‐GENE+ v1.1.6.2 (https://github.com/saigegit/SAIGE), PLINK v2.0 (https://www.cog‐genomics.org/plink/2.0/), SnpEff v5.1 (https://pcingola.github.io/SnpEff/se_introduction/), ANNOVAR (https://annovar.openbioinformatics.org/en/latest/), and BHR v0.1.0 (https://github.com/ajaynadig/bhr).
