# Supplementary material for: Large‐scale Whole‐Exome Sequencing Defines the Protein‐Coding Architecture of Retinal Structure, Visual Function, and Major Blinding Diseases
Source: Adv Sci (Weinh). 2026 Jul 24;13(56):e76582. doi: 10.1002/advs.76582 (PMC13397823; doi:10.1002/advs.76582)
Supplement: Supplementary file 1 [file ADVS-13-e76582-s001.docx]

Supplementary Fig. 1 Quantile-quantile (QQ) plots of gene-based rare-variant association tests.

QQ plots showing the observed versus expected -log_10_(P) values under different maximum minor allele frequency (MAF) thresholds (1×10^-5^, 1×10^-4^, 1×10^-3^, and 1×10^-2^) for protein loss-of-function (pLoF, top) and pLoF plus predicted deleterious missense (pLoF+pmis, bottom) variants. The y-axis represents the observed -log_10_(P) values, and the x-axis the expected distribution under the null hypothesis. The genomic inflation factor (λ) and the number of significant hits (P < 2.5 × 10^-6^) are indicated in each panel. The red dashed line marks the exome-wide significance threshold.

Supplementary Fig. 2 Leave-one-variant-out (LOVO) analyses of significant genes.

LOVO analyses for all gene-phenotype pairs surpassing the exome-wide significance threshold in rare-variant association tests. Each plot shows the gene-phenotype association signal after sequentially removing individual variants. The x-axis denotes the variants tested in the LOVO procedure, and the y-axis shows the resulting gene-level -log_10_(P) values from SAIGE-GENE+ after exclusion of each variant. Red points highlight variants whose removal caused the strongest attenuation of the association signal (P > 0.001), indicating potential driver variants. The horizontal red line represents the original gene-level -log_10_(P) value before LOVO exclusion.

Supplementary Fig. 3 Burden heritability of rare variants across retinal phenotypes.

**A.** Distribution of burden heritability estimates (h^2^, rare) stratified by functional annotation category: predicted loss-of-function (LoF), missense, and LoF+missense variants. Boxplots are overlaid on violin plots to show the spread and median of estimates. **B**. Burden heritability (h^2^, rare) across 16 retinal phenotypes, including 12 OCT structural traits, visual acuity measured by the logarithm of the minimum angle of resolution (logMAR VA), age-related macular degeneration (AMD), glaucoma, and diabetic retinopathy (DR). Each point represents the estimate under a given functional annotation category.

Supplementary Fig. 4 Functional annotation and predicted pathogenicity of significant rare-variant genes

**A.** Gene Ontology (GO) enrichment analysis of biological processes (BP), cellular components (CC), and molecular functions (MF) associated with the significant genes. Top enriched terms include visual system development, photoreceptor differentiation, complement activation, and actin cytoskeleton organization. **B.** AlphaMissense-predicted pathogenicity scores for protein-coding variants in the significant genes. Each point represents a variant predicted to be deleterious, with color indicating the associated retinal phenotype and the x-axis denoting the AlphaMissense score. Variants with scores > 0.8 were classified as high-confidence deleterious.
